# Validating clinical assumptions for characterizing prostate cancer aggressiveness utilizing historical cancer registry data

**DOI:** 10.1093/aje/kwag083

**Published:** 2026-04-14

**Authors:** Kaitlyn M Tsuruda, Hilde Langseth, Lauren M Hurwitz, Stella Koutros, Trude E Robsahm, Rune Kvåle

**Affiliations:** Department of Research, Cancer Registry of Norway, Norwegian Institute of Public Health, 0304 Oslo, Norway; Department of Research, Cancer Registry of Norway, Norwegian Institute of Public Health, 0304 Oslo, Norway; Department of Epidemiology and Biostatistics, School of Public Health, Imperial College London, London, W12 0BZ, United Kingdom; Division of Cancer Epidemiology and Genetics, National Cancer Institute, National Institutes of Health, Bethesda, MD 20892, United States; Division of Cancer Epidemiology and Genetics, National Cancer Institute, National Institutes of Health, Bethesda, MD 20892, United States; Department of Research, Cancer Registry of Norway, Norwegian Institute of Public Health, 0304 Oslo, Norway; Department of Research, Cancer Registry of Norway, Norwegian Institute of Public Health, 0304 Oslo, Norway; Cancer Clinic, Haukeland University Hospital, Helse Bergen HF, 5021 Bergen, Norway

**Keywords:** prostate cancer, aggressive prostate cancer, validation study, population-based cohort, registry data

## Abstract

**The prognosis of prostate cancer is highly dependent on disease characteristics at diagnosis, and dilution effects can compromise etiological studies that are not stratified by disease aggressiveness. However, such classifications are often hindered by missing staging data. This study aimed to develop and validate clinical assumptions to consolidate information about clinical TNM stage and Gleason score (GS) for improved classification of aggressive and non-aggressive disease. A total of 17 551 males diagnosed with prostate cancer (1974-2022) in the Norwegian Janus Serum Bank cohort were included. The developed clinical assumptions were validated using positive predictive values (PPVs) among males with non-missing data and long-term cancer-specific mortality. Cases were classified as aggressive (cT4, N1, M1, or GS ≥ 8) or non-aggressive (complete information without aggressive features) with and without applying the clinical assumptions. Of the 14 developed clinical assumptions, 8 had a PPV** > **95%. Applying these assumptions nearly halved the proportion of cases with unclassifiable aggressivity (from 59% to 32%). Fifteen-year cumulative mortality [95%CI] was higher for previously unclassifiable cases newly classified as aggressive (53.4% [47.6-59.7]) than for cases originally classified as aggressive (42.6% [40.5-44.8]). Clinical assumptions to consolidate TNM and GS information can substantially improve the classification of aggressive and non-aggressive prostate cancer.**

## Introduction

The prognosis of prostate cancer is highly dependent on disease characteristics at diagnosis. The introduction of prostate-specific antigen (PSA) as an early diagnostic tool in the 1990s increased the proportion of patients diagnosed with indolent disease, which may have little impact on life expectancy.^1^^,^^2^ Consequently, many males diagnosed with prostate cancer in the PSA era exhibit a similar life expectancy to the general male population. Etiological studies including patient populations that are not stratified by prostate cancer aggressiveness may therefore be compromised by dilution effects, which can hamper the identification of risk factors for clinically relevant disease.^3^

As part of the National Cancer Institute Cohort Consortium, the Prostate Cancer Cohort Consortium (PC3) unites researchers and resources to investigate prostate cancer etiology, prevention, and survivorship. In 2021, the PC3 recommended definitions of aggressive prostate cancer for etiological epidemiological research based on US data.^4^ These were later applied and validated in the Norwegian Janus Serum Bank (JSB) cohort,^5^ providing an excellent opportunity to study lifestyle-based risk factors in a cohort with over 17 000 incident prostate cancer cases. However, as with many cohorts, the JSB is affected by a substantial amount of missing historical information on important prognostic factors for etiological research. This is particularly the case for Gleason score (GS), for which a consensus statement was not published until after 2000.^6^^,^^7^

Applying validated clinical assumptions to consolidate information about TNM stage and GS from other high-quality variables captured over a long period can enhance the quality and completeness of epidemiological analyses. Moreover, the development of such data consolidation methods may help discriminate aggressive and non-aggressive disease in other national or international studies, particularly those using historical datasets with less comprehensive data collection than contemporary cohorts. Lastly, by better capturing all available information, these data consolidation methods can better position researchers for handling missing data prior to applying statistical methods such as multiple imputation.^8^

This study aimed to develop and validate clinical assumptions to consolidate information about TNM stage and GS for improved classification of aggressive and non-aggressive prostate cancer in etiological studies.

## Methods

### Data sources

#### The Janus Serum Bank and cohort

The JSB is a population-based prospective biobank with blood samples from >318 000 Norwegians. The JSB cohort consists of 152 567 males and 140 284 females recruited from Norwegian health surveys performed during 1972-2003 and Red Cross blood donors from Oslo and surrounding areas.^9^^,^^10^

#### The Cancer Registry of Norway

Prostate cancer information was identified through a deterministic linkage with the Cancer Registry of Norway (CRN) at the Norwegian Institute of Public Health. Our project included diagnoses until December 31, 2022. We included JSB cohort participants diagnosed with prostate cancer (ICD-10 code C61) that was histologically verified as adenocarcinoma (defined in Appendix S1). Subjects were excluded if their prostate cancer diagnosis was prior to their entry into the JSB cohort or their diagnosis was registered based on an autopsy or death certificate only.

The CRN was established in the 1950s with mandatory reporting of cancer diagnoses. Information about morphology has been collected since 1970.^11^ Among prostate cancers diagnosed during 2001-2005, ≥99.7% were registered at the CRN (0.9% from death certificate only) and 97% were morphologically verified.^12^ Estimated completeness is also ≥99% for cases diagnosed before this period.^11^ An investigation of 298 randomly selected prostate cancer registrations during 1957-1986 found an error rate of roughly ≤ 1% per variable for classifications of histology, metastasis, basis of diagnosis, and treatment (including surgery).^11^

Since 2004, there has also been mandatory reporting to the Norwegian Prostate Cancer Registry (NoPCR) at the CRN. This population-based registry contains clinical information including PSA, GS and grade group, clinical and pathological staging, and primary treatment.^13^ Coverage and data quality in the NoPCR have improved over time, and clinical assessments were available for >90% of prostate cancers diagnosed in 2023.^14^ However, data quality for individual variables varies. For example, during 2004-2010, 2%-7% of cases were reported as cTX/cT-missing, vs 76%-82% for cNX/cN-missing.^15^

### Clinical variables

Patient variables included birth year, age at enrollment in the JSB cohort, and age at prostate cancer diagnosis. Disease variables included: basis for diagnosis (histology from primary tumor, histological examination with immunophenotyping, histology from metastasis, other), clinical TNM stage at diagnosis, GS and International Society of Urological Pathology (ISUP) grade group at diagnosis (from biopsy), World Health Organization (WHO) histologic grade (1: well differentiated, 2: moderately differentiated, 3: poorly differentiated, 4: anaplastic), PSA at diagnosis, and a variable for extent of disease defined by the CRN (Table S1). Prostate cancers were classified as low risk (yes/no) using a modified guidelines classification: PSA < 10 ng/mL and GS ≤ 6 (or WHO grade 1, if missing GS) and cT1-2a.^16^^,^^17^ We note that clinical N-stage is determined based on radiological findings. Clinical M-stage is typically determined from bone scintigraphy, magnetic resonance imaging (MRI), or computed tomography (CT). In more recent years, prostate-specific membrane antigen positron emission tomography (PSMA-PET) has also been used. Clinical assessments alone are rarely used to determine M stage.

Information was also available on the most comprehensive primary surgery (none, radical prostatectomy, other), but we were only able to distinguish between radical prostatectomy and cystoprostatectomy from 2008 onwards. For patients diagnosed ≤ 2007, we were only able to identify treatment with radical prostatectomy among those with no history of bladder cancer.

Follow-up information about vital status, including cause of death, was available until June 30, 2024.

### Classification of aggressive prostate cancer

Aggressive prostate cancer was defined using a definition proposed by the PC3 for epidemiological etiological research.^4^ In short, any case with clinical T4, N1, or M1 disease, or with a GS ≥ 8 from a diagnostic biopsy, was classified as “aggressive.” Cases were otherwise classified as “non-aggressive” if they had complete information on all 4 features. Cases were “unclassifiable” if missing data prevented us from making a classification.

### Clinical assumptions for missing data

We developed 14 clinical assumptions based on national coding guidelines, published literature, and international treatment guidelines to deterministically infer values for missing cTNM stage or GS under certain conditions (Table 1).

**Table 1:** Clinical assumptions to infer values for missing cTNM stage or Gleason score (GS).

**Missing variable**	**Clinical assumption**	**Supporting reference(s)**
**cT** ^**a**^	Extent of disease^b^ indicates macroscopic tumor growth into adjacent tissue ⇒ cT4	Cancer Registry of Norway^18^
	Extent of disease^b^ indicates microscopic tumor growth into adjacent tissue ⇒ cT < 4	Cancer Registry of Norway^18^
**cN** ^**a**^	Extent of disease^b^ indicates metastasis to regional lymph nodes ⇒ cN1	Cancer Registry of Norway^18^
	Low-risk prostate cancer^c^ ⇒ cN0	Eifler et al.^19^
	Extent of disease^b^ indicates no metastasis ⇒ cN0	Cancer Registry of Norway^18^
**cM** ^**a**^	Histology from metastasis used as basis for diagnosis ⇒ cM1	Cancer Registry of Norway^20^
	Extent of disease^b^ indicates distant lymph node or organ metastasis ⇒ cM1	Cancer Registry of Norway^18^
	Extent of disease^b^ indicates no metastasis ⇒ cM0	Cancer Registry of Norway^18^
	Prostatectomy as most comprehensive primary surgery ⇒ cM0	NCCN Guidelines^17^; European Association of Urology Guidelines^16^; Norwegian National Guidelines^21^
	Prostate-specific antigen > 100 ng/mL at diagnosis ⇒ cM1	Lojanapiwat et al.^22^; Bjerner et al.^23^; Luining et al.^24^
	Low-risk prostate cancer^c^ ⇒ cM0	Westerberg et al.^25^
**GS**	Histologic grade 3 ⇒ GS ≥ 8	Bjerner et al.^23^
	Histologic grade 2 ⇒ GS < 8	Bjerner et al.^23^
	Histologic grade 1 ⇒ GS < 8	Bjerner et al.^23^

a cTX, cNX, and cMX were considered missing.

b The Cancer Registry of Norway “extent of disease” variable described in Table S1.

c Modified^*^ National Comprehensive Cancer Network (NCCN)/European Association of Urology guidelines classification: (Prostate-specific antigen < 10 ng/mL) and (GS ≤ 6 or World Health Organization histologic grade 1^*^) and (cT1-2a).

### Statistical analysis

Patient and disease characteristics at diagnosis, information on surgical treatment, and vital status at the end of follow-up were described using medians and interquartile ranges (IQRs) or frequencies and proportions, stratified by diagnostic period (≤1989: pre-PSA era in Norway; 1990-2003: prior to the NoPCR; ≥2004: after the NoPCR). Median follow-up after diagnosis was estimated using the reverse Kaplan–Meier estimator.^26^

Clinical assumptions were validated using the subset of the study cohort for whom information was available for the characteristic being determined. Our primary validation metric was the positive predictive value (PPV_inferred_), calculated as the number of correctly classified cases (true positives) divided by the total number of cases classified by the clinical assumption. We considered a PPV of >95% sufficient to demonstrate that a clinical assumption captured information equivalent to the characteristic of interest, while allowing for a small amount of measurement error. An ad hoc sensitivity analysis of PPV_inferred_ was conducted using contemporary cases diagnosed during 2013-2022.

Using the PPV, we also validated the ability of our clinical assumptions to correctly classify a characteristic indicating aggressive prostate cancer (PC3 definition). PPV_PC3_ was calculated as the number of correctly classified aggressive cases (true positives) divided by the total number of cases classified as aggressive by the clinical assumption.

We compared the cumulative prostate cancer-specific mortality after diagnosis for patients with known clinical information for a given characteristic and those with a characteristic classified using a clinical assumption (including patients with a known cTNM or GS). We also estimated cumulative prostate cancer-specific mortality stratified by aggressivity classification. These analyses were performed using the original data and using (previously unclassifiable) cases after applying the clinical assumptions with PPV_inferred_ > 95%. Results were reported 5 and 15 years after diagnosis with corresponding 95% confidence intervals (95% CI). An ad hoc sensitivity analysis was also conducted using cases diagnosed during 2013-2022 after applying clinical assumptions with PPV_inferred_ > 95%.

Lastly, we quantified the frequency and proportion of cases with concordant and discordant classifications from overlapping clinical assumptions (see Appendix S2 for details).

We contextualized the impact of our clinical assumptions by describing their ability to complete missing data based on the set of assumptions with PPV_inferred_ > 95%. This was done for cT, cN, cM, and GS separately and for the aggressivity classifications.

Analyses were performed using Stata 19.5; the *stcrprep* package was used for estimating cumulative prostate cancer-specific mortality.^27^

## Results

**Figure 1:**
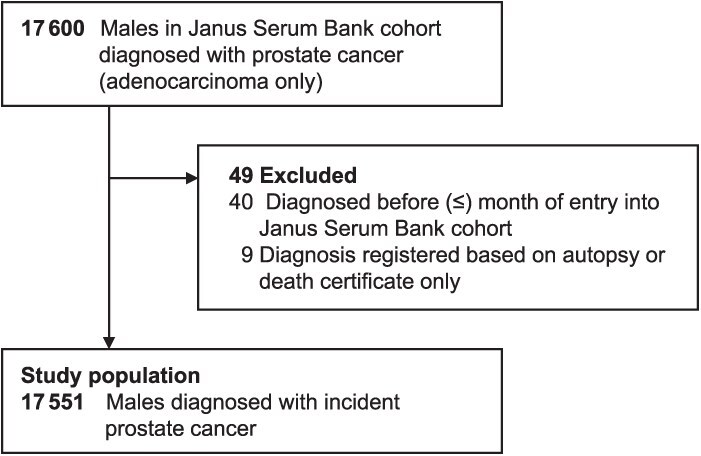
Flow chart illustrating inclusion of study subjects. Exclusion criteria were applied sequentially.

This study included 17 551 males diagnosed with incident prostate cancer between 1974 and 2022 (Figure 1). The median age at enrollment into the JSB cohort was 41 years (range 17 to 87), and the median age at diagnosis was 69 (IQR: 64 to 73) (Table 2). Median follow-up after diagnosis was 11 years.

**Table 2:** Patient and disease characteristics of males diagnosed with prostate cancer in the Janus Serum Bank (JSB) cohort during 1974-2022, *n* (%) unless otherwise specified.

	**Diagnosis period**			**Total**
	**1974-1989**	**1990-2003**	**2004-2022**	
	***n* = 133**	***n* = 2755**	***n* = 14 663**	***n* = 17 551**
**Year of birth, median [IQR]**	1926 [1924, 1928]	1930 [1926, 1937]	1946 [1942, 1948]	1945 [1936, 1948]
**Age at JSB enrollment, median [IQR]**	47 [45, 48]	44 [41, 48]	41 [40, 42]	41 [40, 42]
**Age at diagnosis, median [IQR]**	59 [56, 61]	67 [61, 71]	69 [65, 74]	69 [64, 73]
**Basis for diagnosis**				
Histology from primary tumor	113 (85.0%)	2382 (86.5%)	11 107 (75.7%)	13 602 (77.5%)
Histologic examination with immunophenotyping	0 (0.0%)	201 (7.3%)	3381 (23.1%)	3582 (20.4%)
Histology from metastasis	4 (3.0%)	23 (0.8%)	53 (0.4%)	80 (0.5%)
Other	16 (12.0%)	149 (5.4%)	122 (0.8%)	287 (1.6%)
**Clinical T-stage**				
cT0	0 (0.0%)	8 (0.3%)	60 (0.4%)	68 (0.4%)
cT1	0 (0.0%)	405 (14.7%)	4326 (29.5%)	4731 (27.0%)
cT2	0 (0.0%)	497 (18.0%)	4951 (33.8%)	5448 (31.0%)
cT3	0 (0.0%)	626 (22.7%)	3120 (21.3%)	3746 (21.3%)
cT4	0 (0.0%)	93 (3.4%)	466 (3.2%)	559 (3.2%)
Missing cT or cTX	133 (100.0%)	1126 (40.9%)	1740 (11.9%)	2999 (17.1%)
**Clinical N-stage**				
cN0	0 (0.0%)	528 (19.2%)	5244 (35.8%)	5772 (32.9%)
cN1	0 (0.0%)	42 (1.5%)	667 (4.5%)	709 (4.0%)
Missing cN or cNX	133 (100.0%)	2185 (79.3%)	8752 (59.7%)	11 070 (63.1%)
**Clinical M-stage**				
cM0	0 (0.0%)	877 (31.8%)	6699 (45.7%)	7576 (43.2%)
cM1	0 (0.0%)	124 (4.5%)	943 (6.4%)	1067 (6.1%)
Missing cM or cMX	133 (100.0%)	1754 (63.7%)	7021 (47.9%)	8908 (50.8%)
**ISUP grade group at diagnosis**				
Grade group 1 (GS ≤ 6)	0 (0.0%)	40 (1.5%)	4241 (28.9%)	4281 (24.4%)
Grade group 2 (GS 7a)	0 (0.0%)	21 (0.8%)	3738 (25.5%)	3759 (21.4%)
Grade group 3 (GS 7b)	0 (0.0%)	5 (0.2%)	2444 (16.7%)	2449 (14.0%)
Grade group 4 (GS 8)	0 (0.0%)	7 (0.3%)	2044 (13.9%)	2051 (11.7%)
Grade group 5 (GS 9-10)	0 (0.0%)	8 (0.3%)	1530 (10.4%)	1538 (8.8%)
Unknown/missing	133 (100.0%)	2674 (97.1%)	666 (4.5%)	3473 (19.8%)
**WHO histologic grade**				
Well differentiated (1)	16 (12.0%)	523 (19.0%)	1099 (7.5%)	1638 (9.3%)
Moderately differentiated (2)	45 (33.8%)	901 (32.7%)	2266 (15.5%)	3212 (18.3%)
Poorly differentiated (3)	33 (24.8%)	444 (16.1%)	1473 (10.0%)	1950 (11.1%)
Anaplastic (4)	0 (0.0%)	7 (0.3%)	7 (0.0%)	14 (0.1%)
Unknown/missing	39 (29.3%)	880 (31.9%)	9818 (67.0%)	10 737 (61.2%)
**PSA at diagnosis, ng/mL**				
Median [IQR]		13.3 [7.8, 33.5]	9.5 [6.5, 17.0]	9.5 [6.5, 17.0]
Unknown/missing	133 (100.0%)	2663 (96.7%)	1186 (8.1%)	3982 (22.7%)
**Low risk disease at diagnosis** ^**a**^				
Yes	0 (0.0%)	9 (0.3%)	2052 (14.0%)	2061 (11.7%)
Unknown	133 (100.0%)	1109 (40.3%)	799 (5.4%)	2041 (11.6%)
**Extent of disease**				
No metastasis	78 (58.6%)	1165 (42.3%)	7313 (49.9%)	8556 (48.7%)
Metastasis to regional lymph nodes	5 (3.8%)	79 (2.9%)	876 (6.0%)	960 (5.5%)
Distant lymph node or organ metastasis	36 (27.1%)	297 (10.8%)	961 (6.6%)	1294 (7.4%)
Other	6 (4.5%)	130 (4.7%)	2768 (18.9%)	2904 (16.5%)
Unknown/missing	8 (6.0%)	1084 (39.3%)	2745 (18.7%)	3837 (21.9%)
**Most comprehensive primary surgery** ^**b**^				
None	1 (0.8%)	11 (0.4%)	18 (0.1%)	30 (0.2%)
Radial prostatectomy	12 (9.0%)	401 (14.6%)	4795 (32.7%)	5208 (29.7%)
Other	104 (78.2%)	2294 (83.3%)	9828 (67.0%)	12 226 (69.7%)
Unknown/missing	16 (12.0%)	49 (1.8%)	22 (0.2%)	87 (0.5%)
**Vital status at end of follow-up**				
Alive	0 (0.0%)	427 (15.5%)	10 039 (68.5%)	10 466 (59.6%)
Emigrated or lost to follow-up	0 (0.0%)	4 (0.1%)	17 (0.1%)	21 (0.1%)
Died of cancer	100 (75.2%)	1458 (52.9%)	2751 (18.8%)	4312 (24.6%)
Died of other causes	33 (24.8%)	832 (30.2%)	1628 (11.1%)	2493 (14.2%)
Died, missing cause of death	0 (0.0%)	34 (1.2%)	225 (1.5%)	259 (1.5%)

Abbreviations: GS, Gleason score; IQR, interquartile range; ISUP, International Society of Urological Pathology; PSA, prostate-specific antigen; WHO, World Health Organization.

a Modified^*^ National Comprehensive Cancer Network/European Association of Urology guidelines classification: (prostate-specific antigen < 10 ng/mL) and (GS ≤ 6 or World Health Organization histologic grade 1^*^) and (cT1-2a).

b Surgery may have been performed outside the diagnosis period.

### Aggressivity classification before applying clinical assumptions

**Figure 2:**
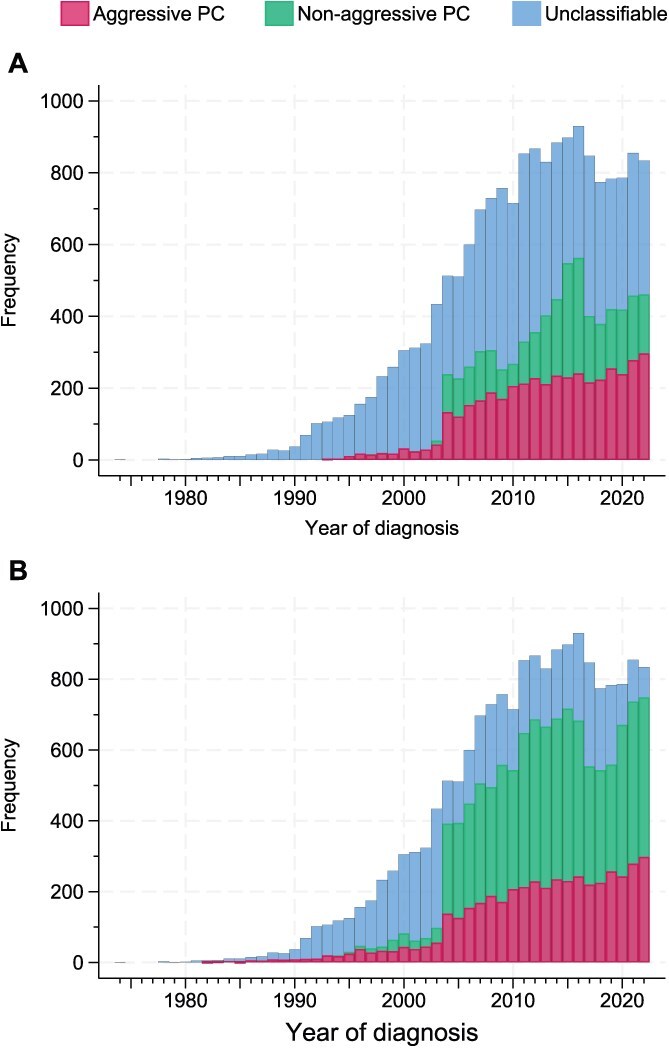
Annual number of prostate cancers (PC) diagnosed among males in the Janus Serum Bank cohort classified as aggressive, non-aggressive, or unclassifiable (A) prior to applying clinical assumptions, and after applying (B) clinical assumptions with PPV_inferred_ > 95%; *n* = 17 551.

Using the definition proposed by the PC3, missing data prevented aggressivity classification in 59% of cases, while 24% were classified as aggressive and 17% as non-aggressive (Figure 2). Overall, over half of the cases were missing information about cN and cM, while roughly 1 in 5 cases were missing information about cT and GS (Table 2). Information about cTNM was first collected in the early 1990s, and completeness for these variables improved with the establishment of NoPCR in 2004 (Figure S1). Missing GS information largely precluded classifying cases as non-aggressive prior to 2004.

There were more unclassifiable cases from the early study period, and these had a very high proportion of missing information (≥75%) about cN and cM (Table S2). Among those with known information on PSA, the median PSA for unclassifiable cases was similar to that of non-aggressive cases (8.2 and 8.9 ng/mL, vs 18.6 ng/mL for aggressive cases).

### Aggressivity classification after applying clinical assumptions

Applying the clinical assumptions with a PPV_inferred_ > 95% facilitated the classification of 4612 prostate cancers, leaving 5668 (32%) unclassifiable (Table 3). This data consolidation improved data completeness in the cTNM and GS variables more in recent years compared to earlier years (Figure 2B, Figure S1).

**Table 3:** Distribution of key variables and classifications of aggressive prostate cancer in the Janus Serum Bank cohort before and after applying the clinical assumptions validated in this study with PPV_inferred_ > 95%, *n* = 17 551.

	**Original data**	**After applying clinical assumptions with PPV**_**inferred**_ **> 95%**
**Clinical T-stage**		
cT0-cT3	13 993 (79.7%)	14 181 (80.8%)
cT4	559 (3.2%)	559 (3.2%)
Missing cT or cTX	2999 (17.1%)	2811 (16.0%)
**Clinical N-stage**		
cN0	5772 (32.9%)	11 224 (64.0%)
cN1	709 (4.0%)	709 (4.0%)
Missing cN or cNX	11 070 (63.1%)	5618 (32.0%)
**Clinical M-stage**		
cM0	7576 (43.2%)	13 334 (76.0%)
cM1	1067 (6.1%)	1371 (7.8%)
Missing cM or cMX	8908 (50.8%)	2846 (16.2%)
**ISUP grade group at diagnosis (biopsy)**		
Grade group 1-3 (GS ≤ 7b)	10 489 (59.8%)	11 093 (63.2%)
Grade group 4-5 (GS ≥ 8)	3589 (20.4%)	3589 (20.4%)
Unknown/missing	3473 (19.8%)	2869 (16.3%)
**PC3 classification**		
Aggressive	4220 (24.0%)	4478 (25.5%)
Non-aggressive	3051 (17.4%)	7405 (42.2%)
Unable to classify	10 280 (58.6%)	5668 (32.3%)

Abbreviations: GS, Gleason score; ISUP, International Society of Urological Pathology; PC3, Prostate Cancer Cohort Consortium; PPV, positive predictive value.

Most (75%) of the cases classified as aggressive after applying our clinical assumptions were diagnosed ≤2001. These cases had a high proportion of missing clinical information but were characterized as having distant metastasis from the Cancer Registry’s extent of disease variable. The remaining unclassifiable cases generally exhibited clinical characteristics similar to those of non-aggressive cases but were diagnosed earlier in the study period and had substantially more missing information about GS.

### Validity of clinical assumptions

#### Positive predictive values

Overall, the PPV_inferred_ ranged from 55.8% to 99.8% (Table 4). Most clinical assumptions to determine cM status had a PPV_inferred_ ≥ 97.5%; however, the clinical assumption that a PSA > 100 ng/mL represented cM1 status had a PPV_inferred_ of 72.7%. When determining GS, the PPV_inferred_ was highest (98.7%) for the assumption that well-differentiated (grade 1) disease indicated a GS < 8 and lowest (85.8%) for the assumption that poorly differentiated (grade 3) disease indicated a GS ≥ 8. Our clinical assumption for identifying cT4 disease had a substantially lower PPV_inferred_ than that for identifying <cT4 disease (55.8% vs 99.6%). Similarly, our clinical assumption for identifying cN1 disease also had a lower PPV_inferred_ than those for identifying cN0 disease (63.7% vs 99.8%). The PPV_inferred_ for contemporary cases diagnosed during 2013-2022 (*n* = 8421) were generally similar to those observed for the whole cohort (Table S3).

**Table 4:** Validation results for clinical assumptions to derive information for missing cTNM stage or Gleason score (GS).

**Missing feature**	**Clinical assumption**	**Values inferred from clinical assumption, *n*** ^***a***^	**Validity against known clinical feature**		**Validity against known aggressive prostate cancer**	
			***n***	**PPV**_**inferred**_	***n***	**PPV**_**PC3**_
cT^**b**^	Extent of disease^c^ indicates macroscopic tumor growth into adjacent tissue ⇒ cT4	22	147	55.8%	110	90.0%
	Extent of disease^c^ indicates microscopic tumor growth into adjacent tissue ⇒ cT < 4	188	2513	99.6%		
cN^**b**^	Extent of disease^c^ indicates metastasis to regional lymph nodes ⇒ cN1	472	488	63.7%	716	92.9%
	Low-risk prostate cancer^d^ ⇒ cN0	1437	624	99.8%		
	Extent of disease^c^ indicates no metastasis ⇒ cN0	5071	3485	99.8%		
cM^**b**^	Histology from metastasis used as basis for diagnosis ⇒ cM1	42	38	89.5%	42	100%
	Extent of disease^c^ indicates distant lymph node or organ metastasis ⇒ cM1	304	990	97.5%	1027	99.9%
	Extent of disease^c^ indicates no metastasis ⇒ cM0	4359	4197	99.5%		
	Prostatectomy as most comprehensive primary surgery ⇒ cM0	2556	2652	99.5%		
	Prostate-specific antigen > 100 ng/mL at diagnosis ⇒ cM1	106	549	72.7%	593	98.7%
	Low-risk prostate cancer^d^ ⇒ cM0	1257	804	99.8%		
GS	Histologic grade 3 ⇒ GS ≥ 8	517	1433	85.8%	1402	95.9%
	Histologic grade 2 ⇒ GS < 8	980	2232	92.7%		
	Histologic grade 1 ⇒ GS < 8	604	1034	98.7%		

a Based on filling in only the missing value assessed by the clinical assumption (ie, not a combination of assumptions). Note that identifying a single aggressive feature is sufficient to classify a cancer as aggressive, while complete information on all features is needed to classify a case as non-aggressive.

b cTX, cNX, and cMX were considered missing.

c The Cancer Registry of Norway “extent of disease” variable is described in Table S1.

d Modified^*^ National Comprehensive Cancer Network/European Association of Urology guidelines classification: (prostate-specific antigen < 10 ng/mL) and (GS ≤ 6 or World Health Organization histologic grade 1^*^) and (cT1-2a).

The PPV_PC3_ ranged from 90% to 100%, and 4 clinical assumptions had a PPV_PC3_ > 95% (Table 4).

#### Cumulative prostate cancer-specific mortality

**Figure 3:**
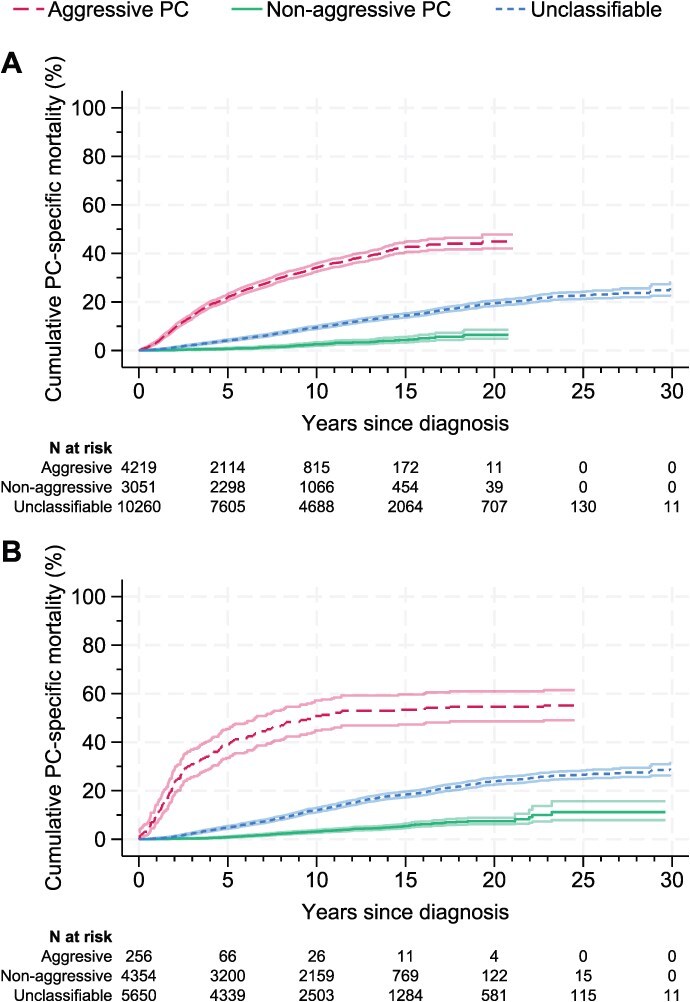
Cumulative prostate cancer-specific mortality, stratified by aggressivity classified using (A) the Prostate Cancer Cohort Consortium (PC3) definition, and (B) among unclassifiable prostate cancer (PC) cases shown in panel A after applying clinical assumptions with PPV_inferred_ > 95%.

The 15 year cumulative prostate cancer-specific mortality was notably higher for aggressive cases (42.6%) than for non-aggressive cases (4.3%) (Figure 3A). When the clinical assumptions with a PPV_inferred_ > 95% were applied to previously unclassifiable cases, the 15 year cumulative prostate cancer-specific mortality was 53.4% for aggressive cases and 5.5% for non-aggressive cases (Figure 3B). Full results with 95%CI are provided in Table S4.

Among contemporary cases diagnosed during 2013-2022, those originally classified as aggressive had lower year prostate-cancer specific mortality than those diagnosed during the whole study period. Moreover, unclassifiable contemporary cases demonstrated a survival pattern much more consistent with those of non-aggressive cases (Figure S2).

Among patients with known cN0 disease, the 15 year cumulative prostate cancer-specific mortality was 10.4% (Figure S3C). We observed a similar or lower cumulative mortality for patients implied to have cN0 disease using a clinical assumption (Figure S3D and E).

Among patients with known cM1 disease, cumulative prostate cancer-specific mortality increased sharply during the first 5 years after diagnosis and plateaued after roughly 15 years at 72.0% (Figure S4A). Similar trends were observed for all clinical assumptions implying cM1 disease (Figure S4B-D).

Among patients with known cM0 disease, the cumulative prostate cancer-specific mortality increased linearly after diagnosis and was 13.8% after 15 years, plateauing after roughly 23 years (Figure S4E). Patients who received treatment with radical prostatectomy or who had low-risk disease had a lower prostate cancer-specific mortality than those with known cM0 disease (Figure S4G and H).

Cumulative prostate cancer-specific mortality results for all clinical assumptions with corresponding 95%CI are described in Figures S3-S6 and Table S5.

#### Concordance between clinical assumptions

There was > 98% concordance between clinical features for cases that could be assigned a value for cN or cM based on ≥2 clinical assumptions. Details are reported in Appendix S2 and Figures S7 and S8.

## Discussion

The results from the present study provide a strong scientific foundation for consolidating clinical information across multiple variables to improve aggressivity classifications based on TNM stage and GS. After applying our validated clinical assumptions with a PPV_inferred_ > 95%, the proportion of patients with unclassifiable aggressivity was nearly halved. This substantial improvement in disease classification is vital for robust identification of etiological risk factors for prostate cancer. The high PPVs demonstrated by many of our clinical assumptions, and the prognostic discriminative ability demonstrated in our estimates of cumulative prostate cancer-specific mortality, suggest that most of the presented clinical assumptions are valid for data consolidation in epidemiological studies.

Identifying etiological risk factors for aggressive prostate cancer in historical cohorts can be challenging due to a high proportion of missing information about disease aggressiveness when a limited set of key variables (eg, TNM stage and GS) are used. Our analysis focused on the effects of classifying prostate cancer aggressiveness after applying the best-performing clinical assumptions (PPV_inferred_ > 95%). In our view, these clinical assumptions accurately capture information equivalent to the characteristic of interest and represent a method for data consolidation. One third of cases remained unclassifiable in our study after this data consolidation.

The clinical assumptions in our study with a PPV_inferred_ ≤ 95% are less reliable for data consolidation due to increased uncertainty in the resulting classifications. Using statistical methods such as multiple imputation to handle these and other remaining missing data is outside of the scope of our study since such models should be developed for a specific research question and estimand.^28^^,^^29^ Nonetheless, combining deterministic clinical assumptions and multiple imputation has shown promise in addressing data on missing prostate cancer stage.^25^ Our clinical assumptions are useful in this context by helping researchers make better use of available information through data consolidation. Additionally, researchers may consider whether some clinical assumptions with a PPV_inferred_ ≤ 95% could be used as auxiliary variables in imputation models, specifically those that demonstrated an ability to predict the missing features of interest. For example, the association between histology from metastasis used as the basis for a prostate cancer diagnosis and the presence of distant metastasis at diagnosis was insufficient for data consolidation (PPV_inferred_ = 89.5%) but may still be informative in an imputation model.

Detailed diagnostic information, including cTNM stage, GS, PSA at diagnosis, and primary treatment, was routinely collected after the NoPCR was established, which reduced the proportion of missing data in our study.^13^ As such, the benefits of our clinical assumptions were greater for cases diagnosed after 2004 than before. Nonetheless, we remained unable to determine aggressivity for 22% of cases after 2004, and N-stage was missing for 75% of these cases. cNX is often reported because lymph nodes have not been examined by relevant imaging or no N-category is given, even when there is a low risk of node involvement. Missing N-stage was also the primary reason for missing staging in the Danish Cancer Registry database from 2004 to 2009.^30^ The small prognostic difference between cases with missing or complete information on N-status among non-distant metastatic cases with <cT3 and GS < 8 indicates that most cancers with low- or intermediate-risk disease characteristics with NX or N-missing are non-aggressive (Figure S9). However, our conservative approach only reclassifies NX/N-missing as N0 for cases defined as low risk using a modified guidelines classification, in which the reported risk of lymph-node metastases is <1%^19^ and the observed PPV in our study was 99.8%.

Previous studies have demonstrated that assuming M0 for cases with unknown M status underestimates the incidence of M1 disease.^25^^,^^31^ Our validated assumptions for inferring cM status reduced missing M status from every second case to roughly every sixth case. These assumptions all had a PPV_inferred_ of about 90% or higher, except for the assumption that PSA > 100 ng/mL implies M1 disease (PPV_inferred_ = 72.7%). Two observational studies reported that a PSA ≥ 100 ng/mL has a PPV of 55%-75% to indicate metastatic prostate cancer based on conventional bone imaging,^32^^,^^33^ while a more recent study demonstrated that this PPV can be as high as 87.5% when contemporary imaging with PSMA-PET is used as the reference standard.^24^ Assuming that a PSA > 100 ng/mL represents a constant biological risk of metastatic disease over time, the PPVs observed in our study are likely biased downward due to the prevalence of conventional imaging used in our historic cohort to establish cM status. The validity of this assumption against a known assessment of aggressive prostate cancer (PPV_PC3_ > 98%) and the high cumulative prostate cancer-specific mortality show that PSA > 100 ng/mL is indeed a good marker for aggressive disease. Nonetheless, the true PPV appears to be < 95%, and we therefore urge researchers to consider PSA as an auxiliary variable in imputation models rather than a direct proxy for cM1 status.

The highest PPVs in our study were associated with clinical assumptions for non-aggressive clinical features (ie, <cT4, cN0, cM0, or GS ≤ 8). This is to some extent expected since, all other factors held equal, PPV will increase if the outcome of interest becomes more prevalent,^34^ and non-aggressive prostate cancer is more prevalent than aggressive prostate cancer. We additionally validated our clinical assumptions using cumulative prostate cancer-specific mortality, which is unaffected by disease prevalence. We observed that the clinical assumptions with the lowest PPVs (ie, for identifying cT4 and cN1 disease) also had limited prognostic discriminative ability. Using assumptions with low PPVs for data consolidation underestimates the uncertainty in any subsequent analyses and can lead to potentially misleading conclusions.

The internal validity of our clinical assumptions was very high, with minimal discordance between assumptions for a given patient. This discordance is unlikely to affect the results of epidemiological studies. We nonetheless recommend that multiple clinical assumptions be applied in an explicit order, with the most reliable applied first. In our paper, the clinical assumptions were applied in the order presented in Table 1. Lastly, we note that performing data consolidation to classify disease status at diagnosis based on later treatment information can be useful for etiological studies but may induce immortal time bias in survivorship studies.^35^

A limitation of our study is that we were unable to validate our clinical assumptions on the complete JSB cohort because validation required complete data for the characteristic of interest and the relevant auxiliary variable. PPVs are influenced by disease prevalence,^34^ and patients with missing data were more often diagnosed earlier in the study period when the prevalence of aggressive features was higher compared to more recent years with improved data completeness. Nonetheless, patients diagnosed before 1990 (pre-PSA period) represent 0.8% of the JSB cohort, and the PPV_inferred_ for patients diagnosed during 2013-2022 were generally similar to those observed in our main analysis (particularly for clinical assumptions with a PPV_inferred_ > 95%). This implies that the PPV_inferred_ are likely to be similar for patients diagnosed before 2013, with a few exceptions that can be identified from Table S3. Based on these results and standardized coding rules at the CRN, we believe our PPV results are broadly generalizable to the full JSB cohort. Another limitation of our study is the lack of validation in an external cohort. In general, external validity may vary for different periods and cohorts based on the prevalence of various clinical features and differences in coding. However, our assumptions are largely based on common international clinical characteristics with demonstrated prognostic importance in many different patient populations. In US-based studies, for example, the SEER summary stage is analogous to the CRN’s “extent of disease” variable and could be used to infer values for cTNM. We believe that results from our study can therefore be applied to most prostate cancer populations and time periods and encourage researchers to determine the PPV_inferred_ in their own datasets to confirm the performance of these assumptions prior to any data consolidation.

Our study applied a definition of aggressive prostate cancer intended for etiological epidemiological research and did not consider risk groups for biochemical recurrence defined by current clinical guidelines.^4^^,^^16^^,^^17^ Population-based epidemiological studies typically lack the detailed clinical information needed to implement these definitions (eg, PSA is missing in many epidemiological studies, which often rely on registry linkages to obtain clinical data fields pertaining to cancer diagnosis, particularly for historical cohorts with a long follow-up period^4^). The definition of aggressive prostate cancer used in this study nonetheless overlaps with the NCCN and EAU definitions for high-risk cancer, and because our clinical assumptions are defined for specific clinical factors, those with the highest PPVs can still support data consolidation for research using clinical definitions of high-risk disease. We nonetheless note that the clinical assumptions presented in this study are not intended to guide clinical decisions.

Strengths of our study include the unique opportunity to use information from several high-quality data sources, including prospectively collected population-based clinical data, complete and deterministic registry linkages, and long-term follow-up data with nearly no loss of follow-up. This provided the exceptional possibility to examine, in addition to PPVs, the real-world prognostic importance of our assumptions 15-20 years after diagnosis. The fact that the prognostic difference between patients categorized as aggressive and non-aggressive was greater in cases classified after applying our clinical assumptions than in the original data is also reassuring. Although the prognostic difference between patients categorized as aggressive and non-aggressive decreased in contemporary cases, there was still a notable difference in mortality between these groups.

## Conclusion

Utilizing selected clinical assumptions based on available cancer registry information to consolidate information about TNM and GS can substantially improve the classification of aggressive and non-aggressive diseases. This major advancement in disease classification is crucial for identifying etiological risk factors for prostate cancer.

## Ethics approval and compliance with ethical standards

This study was approved by the Regional Committees for Medical Research Ethics, South East Norway (approval number 22858, previously 2014/204). Janus Serum Bank participants have given a broad consent for the use of their samples and data in cancer research. This study was performed in compliance with all relevant laws and institutional guidelines and was performed in accordance with the Declaration of Helsinki.

## Supplementary Material

Web_Material_kwag083

## Data Availability

The data can be shared for research purposes on request to the Cancer Registry of Norway’s data delivery unit via Helsedata.no (https://helsedata.no/en/). Access is conditional on adherence to local ethical and security policies. Further information, including the code used to conduct specific analyses, is available from the authors upon request.
