# Supplementary material for: Validating clinical assumptions for characterizing prostate cancer aggressiveness utilizing historical cancer registry data
Source: Am J Epidemiol. 2026 Apr 14;195(8):2297–306. doi: 10.1093/aje/kwag083 (PMC13437071; doi:10.1093/aje/kwag083)
Supplement: Supplementary file 1 [file web_material_kwag083.zip › SUPPLE~1.DOC]

Validating clinical assumptions for characterizing prostate cancer aggressiveness utilizing historical cancer registry data [Supplementary material]

Kaitlyn M Tsuruda^a^, Hilde Langseth^a,b^, Lauren M Hurwitz^c^, Stella Koutros^c^, Trude E Robsahm^a^, Rune Kvåle^a,d^

a) Norwegian Institute of Public Health, Cancer Registry of Norway, Department of Research, P.O. Box 5313 Majorstuen, 0304 Oslo, Norway
b) Imperial College London, School of Public Health, Department of Epidemiology and Biostatistics, White City Campus, 90 Wood Lane, London, W12 0BZ, UK
c) National Institutes of Health, National Cancer Institute, Division of Cancer Epidemiology and Genetics, 9609 Medical Center Drive, MSC 9776, Bethesda, MD, 20892, USA
d) Helse Bergen HF, Haukeland University Hospital, Cancer Clinic, P.O. Box 1400, 5021 Bergen, Norway

**Corresponding author**

Kaitlyn Tsuruda
Department of Research, Cancer Registry of Norway
Norwegian Institute of Public Health
P.O. Box 5313 Majorstuen
0304 Oslo
Norway
[kaitlyn.tsuruda@fhi.no](mailto:kaitlyn.tsuruda@fhi.no)

Supplementary material

[Appendix S1: Definition of adenocarcinoma	3](#_Toc223360815)

[Appendix S2: Internal validity of clinical assumptions	3](#_Toc223360816)

[Methods	3](#_Toc223360817)

[Results	3](#_Toc223360818)

[Tables	4](#_Toc223360819)

[Table S1	4](#_Toc223360820)

[Table S2	5](#_Toc223360821)

[Table S3	6](#_Toc223360822)

[Table S4	7](#_Toc223360823)

[Table S5	8](#_Toc223360824)

[Figures	9](#_Toc223360825)

[Figure S1	9](#_Toc223360826)

[Figure S2	10](#_Toc223360827)

[Figure S3	11](#_Toc223360828)

[Figure S4	12](#_Toc223360829)

[Figure S5	13](#_Toc223360830)

[Figure S6	14](#_Toc223360831)

[Figure S7	15](#_Toc223360832)

[Figure S8	16](#_Toc223360833)

[Figure S9	17](#_Toc223360834)

## Appendix S1: Definition of adenocarcinoma

Prostate cancer was defined as being histologically verified adenocarcinoma if the associated International Classification of Diseases for Oncology, 3rd edition (ICD-O-3) morphology code contained any of the following first four digits: 8140–8149, 8160–8162, 8190–8221, 8260–8337, 8350–8551, 8570–8576, 8940–8941.

## Appendix S2: Internal validity of clinical assumptions

### Methods

We quantified the frequency of cases with concordant and discordant classifications from our clinical assumptions and visualized the results using upset plots. This analysis was performed on all cases, irrespective of whether information was missing on cTNM or Gleason score. However, this analysis was only possible for clinical assumptions regarding cN and cM, as there was no overlap in the cases covered by our assumptions for cT or Gleason score.

### Results

Of the 9979 cases assigned a value for cN based on our clinical assumptions, 1598 (16%) were assigned a value based on 2 different assumptions. Among these 1598, there were only 3 cases with discordant classifications, all of which had been inferred to be cN0 based on the presence of low-risk disease, but also as cN1 based on the extent of disease variable (**Figure S7**).

Of the 12,708 cases assigned a value for cM based on our clinical assumptions, 4773 (38%) were assigned a value based on 2 or 3 different assumptions. There was concordance across clinical assumptions for 99% of these 4773 cases (**Figure S8**). Among the 68 cases with discordant classifications, 45 were inferred to be cM0 based on the extent of disease variable, but also as cM1 due to having a PSA > 100 ng/ml at diagnosis. An additional 12 were inferred to be cM0 based on having received treatment with prostatectomy, but also as cM1 based on the extent of disease variable.

## Tables

### Table S1

Description of the Cancer Registry of Norway’s “extent of disease” variable and its distribution among males diagnosed with prostate cancer in the Janus Serum Bank cohort during 1974–2022 (n = 17,551). Information for this variable may come from clinical and/or pathological sources. A full description of this variable in English and Norwegian can be found online at <https://metadata.kreftregisteret.no/variables/detail/109?tabIndex=1>.

| **Code Value** | **Description** | **Valid from** | **Corresponding SEER stage** | **Clinical assumption** | **n (%)** |
| --- | --- | --- | --- | --- | --- |
| 0 | No metastasis | 1953 | Localised | N0, M0 | 8556 (48.8) |
| 1 | Metastasis to regional lymph nodes | 1986 | Regional | N1 | 958 (5.5) |
| 2 | Metastasis to distant lymph nodes | 1986 | Distant | M1 | 66 (0.4) |
| 3 | Metastasis to organ in the same body region as primary tumor | 1986 | Distant | M1 | 15 (0.1) |
| 4 | Metastasis to organ to another body region than primary tumor | 1986 | Distant | M1 | 1206 (6.9) |
| 5 | Microscopic tumor growth into adjacent tissue | 1986 | Regional | <T4 | 2701 (15.4) |
| 6 | Macroscopic tumor growth into adjacent tissue | 1986 | Regional,  skip metastasis | T4 | 169 (1.0) |
| 7 | Metastasis found, primary tumor unknown | 1986 | Other/unknown | - | 31 (0.2) |
| 8 | Microscopically infiltrating tumor | 1986 | Localised | - | - |
| 9 | Unknown metastasis at time of diagnosis | 1986 | Other/unknown | - | 3837 (21.9) |
| A | Regional lymph node metastasis (clinically or histologically) | 1953 | Regional | N1 | 2 (<0.1) |
| B | Distant lymph node metastasis or organ metastasis | 1953 | Distant metastasis | M1 | 7 (<0.1) |
| C | Metastasis found, primary tumor unknown | 1953 | Other/unknown | - | - |
| D | Direct growth into adjacent tissue or organ | 1953 | Regional | - | 3 (<0.1) |

Table S2

Patient and disease characteristics of males diagnosed with prostate cancer in the Janus Serum Bank (JSB) cohort during 1974–2022 stratified by aggressivity classification before applying clinical assumptions, n (%) unless otherwise specified

|  | Aggressivity classification, original data | | |
| --- | --- | --- | --- |
|  | Aggressive | Not aggressive | Unclassifiable |
|  | n = 4220 | n = 3051 | n = 10,280 |
| Year of birth, median [IQR] | 1945 [1935, 1948] | 1947 [1944, 1949] | 1945 [1932, 1948] |
| Age at JSB enrollment, median [IQR] | 41 [40, 43] | 41 [40, 42] | 41 [40, 43] |
| Age at diagnosis, median [IQR] | 71 [66, 76] | 68 [64, 72] | 68 [63, 72] |
| Year of diagnosis, median [IQR] | 2014 [2009, 2019] | 2015 [2011, 2018] | 2010 [2003, 2016] |
| Basis for diagnosis |  |  |  |
| Histology from primary tumour | 3512 (83.2%) | 2207 (72.3%) | 7883 (76.7%) |
| Histological examination with immunophenotyping | 594 (14.1%) | 829 (27.2%) | 2159 (21.0%) |
| Histology from metastasis | 42 (1.0%) | 0 (0.0%) | 38 (0.4%) |
| Other | 72 (1.7%) | 15 (0.5%) | 200 (1.9%) |
| Clinical T-stage |  |  |  |
| cT0 | 8 (0.2%) | 13 (0.4%) | 47 (0.5%) |
| cT1 | 449 (10.6%) | 895 (29.3%) | 3387 (32.9%) |
| cT2 | 1057 (25.0%) | 1406 (46.1%) | 2985 (29.0%) |
| cT3 | 1725 (40.9%) | 737 (24.2%) | 1284 (12.5%) |
| cT4 | 559 (13.2%) | 0 (0.0%) | 0 (0.0%) |
| Missing cT or cTX | 422 (10.0%) | 0 (0.0%) | 2577 (25.1%) |
| Clinical N-stage |  |  |  |
| cN0 | 1452 (34.4%) | 3051 (100.0%) | 1269 (12.3%) |
| cN1 | 709 (16.8%) | 0 (0.0%) | 0 (0.0%) |
| Missing cN or cNX | 2059 (48.8%) | 0 (0.0%) | 9011 (87.7%) |
| Clinical M-stage |  |  |  |
| cM0 | 2000 (47.4%) | 3051 (100.0%) | 2525 (24.6%) |
| cM1 | 1067 (25.3%) | 0 (0.0%) | 0 (0.0%) |
| Missing cM or cMX | 1153 (27.3%) | 0 (0.0%) | 7755 (75.4%) |
| ISUP grade group at diagnosis |  |  |  |
| Grade group 1 (Gleason Score ≤ 6) | 41 (1.0%) | 1013 (33.2%) | 3227 (31.4%) |
| Grade group 2 (Gleason Score 7a) | 112 (2.7%) | 1152 (37.8%) | 2495 (24.3%) |
| Grade group 3 (Gleason Score 7b) | 183 (4.3%) | 886 (29.0%) | 1380 (13.4%) |
| Grade group 4 (Gleason Score 8) | 2051 (48.6%) | 0 (0.0%) | 0 (0.0%) |
| Grade group 5 (Gleason Score 9–10) | 1538 (36.4%) | 0 (0.0%) | 0 (0.0%) |
| Unknown/missing | 295 (7.0%) | 0 (0.0%) | 3178 (30.9%) |
| WHO histologic grade |  |  |  |
| Well differentiated (1) | 27 (0.6%) | 233 (7.6%) | 1378 (13.4%) |
| Moderately differentiated (2) | 299 (7.1%) | 698 (22.9%) | 2215 (21.5%) |
| Poorly differentiated (3) | 1345 (31.9%) | 57 (1.9%) | 548 (5.3%) |
| Anaplastic (4) | 8 (0.2%) | 1 (0.0%) | 5 (0.0%) |
| Unknown/missing | 2541 (60.2%) | 2062 (67.6%) | 6134 (59.7%) |
| PSA at diagnosis, ng/ml |  |  |  |
| Median [IQR] | 18.6 [9.2, 51.0] | 8.9 [6.3, 13.8] | 8.2 [5.9, 12.0] |
| Unknown/missing | 422 (10.0%) | 26 (0.9%) | 3534 (34.4%) |
| Low risk disease at diagnosis^a^ |  |  |  |
| Yes | 3 (0.1%) | 481 (15.8%) | 1577 (15.3%) |
| Unknown | 160 (3.8%) | 0 (0.0%) | 1881 (18.3%) |
| Extent of disease |  |  |  |
| No metastasis | 932 (22.1%) | 2135 (70.0%) | 5489 (53.4%) |
| Metastasis to regional lymph nodes | 665 (15.8%) | 51 (1.7%) | 244 (2.4%) |
| Distant lymph node or organ metastasis | 1026 (24.3%) | 1 (0.0%) | 267 (2.6%) |
| Other | 1026 (24.3%) | 760 (24.9%) | 1118 (10.9%) |
| Unknown/missing | 571 (13.5%) | 104 (3.4%) | 3162 (30.8%) |
| Most comprehensive primary surgery^b^ |  |  |  |
| None | 13 (0.3%) | 0 (0.0%) | 17 (0.2%) |
| Radial prostatectomy | 920 (21.8%) | 1245 (40.8%) | 3043 (29.6%) |
| Other | 3284 (77.8%) | 1806 (59.2%) | 7136 (69.4%) |
| Unknown/missing | 3 (0.1%) | 0 (0.0%) | 84 (0.8%) |

IQR: Interquartile range, ISUP: International Society of Urological Pathology, PSA: prostate-specific antigen, WHO: World Health Organization
^a^ Modified* National Comprehensive Cancer Network/European Association of Urology guidelines classification: (Prostate-specific antigen < 10 ng/ml) and (GS ≤ 6 or World Health Organization histologic grade 1*) and (cT1-2a)
^b^ Surgery may have been performed outside the diagnosis period

Table S3

Validation results for clinical assumptions to consolidate information about cTNM stage or Gleason score (GS) among contemporary cases diagnosed during 2013–2022, n = 8421.

| Missing feature | Clinical assumption | Validity against known clinical feature | | |
| --- | --- | --- | --- | --- |
|  |  | n | PPV_inferred_ | Δ from Table 4 |
| cT^a^ | Extent of disease^b^ indicates macroscopic tumor growth into adjacent tissue ⇒ cT4 | 65 | 86.2% | +30.4 %pt |
|  | Extent of disease^b^ indicates microscopic tumor growth into adjacent tissue ⇒ cT < 4 | 1801 | 99.9% | +0.3 %pt |
| cN^a^ | Extent of disease^b^ indicates metastasis to regional lymph nodes ⇒ cN1 | 342 | 52.1% | -11.6 %pt |
|  | Low-risk prostate cancer^c^ ⇒ cN0 | 421 | 100% | +0.2 %pt |
|  | Extent of disease^b^ indicates no metastasis ⇒ cN0 | 2144 | 100% | +0.2 %pt |
| cM^a^ | Histology from metastasis used as basis for diagnosis ⇒ cM1 | 23 | 87.0% | -2.5 %pt |
|  | Extent of disease^b^ indicates distant lymph node or organ metastasis ⇒ cM1 | 517 | 98.8% | +1.3 %pt |
|  | Extent of disease^b^ indicates no metastasis ⇒ cM0 | 2221 | 99.9% | +0.4 %pt |
|  | Prostatectomy as most comprehensive primary surgery ⇒ cM0 | 1514 | 99.4% | -0.1 %pt |
|  | Prostate-specific antigen > 100 ng/ml at diagnosis ⇒ cM1 | 253 | 77.5% | +4.8 %pt |
|  | Low-risk prostate cancer^c^ ⇒ cM0 | 445 | 99.8% | No change |
| GS | Histologic grade 3 ⇒ GS ≥ 8 | 1069 | 90.7% | +4.9 %pt |
|  | Histologic grade 2 ⇒ GS < 8 | 1746 | 91.9% | -0.8 %pt |
|  | Histologic grade 1 ⇒ GS < 8 | 729 | 99.3% | +0.6 %pt |

%pt: percentage points  ^a^ cTX, cNX, and cMX were considered missing
^b^ The Cancer Registry of Norway “extent of disease” variable is described in **Table S1**
^c^ Modified* National Comprehensive Cancer Network/European Association of Urology guidelines classification: (Prostate-specific antigen < 10 ng/ml) and (GS ≤ 6 or World Health Organization histologic grade 1*) and (cT1-2a)

Table S4

Cumulative prostate cancer-specific mortality 5 and 15 years after diagnosis with 95% confidence intervals among males in the Janus Serum Bank cohort, stratified by Prostate Cancer Cohort Consortium (PC3) aggressivity classification, assumptions used for data consolidation, and diagnosis period (1974–2022 or 2013–2022).

| **Classification** | **Assumptions used for data consolidation** | **Diagnosis period** | **n at diagnosis** | **Cumulative prostate cancer-specific mortality (%)** | |
| --- | --- | --- | --- | --- | --- |
|  |  |  |  | **5y after diagnosis** | **15y after diagnosis** |
| **Aggressive** | **None (original data)** | **1974–2022** | **4219** | **21.9 (20.6, 23.2)** | **42.6 (40.5, 44.8)** |
|  | Clinical assumptions with PPV_inferred_ > 95% | 1974–2022 | 256 | 39.2 (33.5, 45.5) | 53.4 (47.3, 59.7) |
|  | **None (original data)** | **2013–2022** | **2426** | **16.3 (14.8, 18.0)** | **Not observed** |
|  | Clinical assumptions with PPV_inferred_ > 95% | 2013–2022 | 15 | Not calculated | Not observed |
| **Non-aggressive** | **None (original data)** | **1974–2022** | **3051** | **0.7 (0.5, 1.1)** | **4.3 (3.4, 5.6)** |
|  | Clinical assumptions with PPV_inferred_ > 95% | 1974–2022 | 4354 | 0.9 (0.6, 1.2) | 5.5 (4.6, 6.5) |
|  | **None (original data)** | **2013–2022** | **2074** | **0.7 (0.4, 1.2)** | **Not observed** |
|  | Clinical assumptions with PPV_inferred_ > 95% | 2013–2022 | 2053 | 0.5 (0.3, 1.1) | Not observed |
| **Unknown** | **None (original data)** | **1974–2022** | **10,260** | **4.1 (3.8, 4.6)** | **14.4 (13.6, 15.3)** |
|  | Clinical assumptions with PPV_inferred_ > 95% | 1974–2022 | 5650 | 4.9 (4.3, 5.5) | 18.4 (17.3, 19.6) |
|  | **None (original data)** | **2013–2022** | **3916** | **1.2 (0.9, 1.6)** | **Not observed** |
|  | Clinical assumptions with PPV_inferred_ > 95% | 2013–2022 | 1848 | 1.6 (1.1, 2.3) | Not observed |

Table S5

Cumulative prostate cancer-specific mortality 5 and 15 years after diagnosis with 95% confidence intervals among males in the Janus Serum Bank cohort diagnosed during 1974–2022 (n = 17,551), stratified by known or inferred clinical feature.

| **Known or inferred clinical feature** | **n at diagnosis** | **Cumulative prostate cancer-specific mortality (%)** | |
| --- | --- | --- | --- |
|  |  | **5y after diagnosis** | **15y after diagnosis** |
| **Known cT4** | **559** | **48.5% (44.2, 52.9)** | **67.7% (63.0, 72.4)** |
| cT4 inferred from extent of disease variable | 168 | 16.5% (11.5, 23.2) | 37.9% (29.8, 47.5) |
| **Known <cT4** | **13,993** | **5.5% (5.1, 5.9)** | **15.7% (15.0, 16.5)** |
| <cT4 inferred from extent of disease variable | 2701 | 3.6% (2.9, 4.4) | 13.8% (11.7, 16.3) |
| **Known cN1** | **709** | **34.9% (31.2, 38.9)** | **54.8% (49.9, 59.8)** |
| cN1 inferred from extent of disease variable | 959 | 13.4% (11.3, 15.9) | 36.3% (32.4, 40.5) |
| **Known cN0** | **5772** | **2.8% (2.4, 3.3)** | **10.4% (9.3, 11.7)** |
| cN0 inferred based on low-risk disease at diagnosis | 2061 | 0.5% (0.3, 1.0) | 2.2% (1.5, 3.1) |
| cN0 inferred from extent of disease variable | 8544 | 2.1% (1.8, 2.5) | 10.1% (9.2, 10.9) |
| **Known cM1** | **1067** | **52.5% (49.3, 55.7)** | **72.0% (68.5, 75.5)** |
| cM1 inferred from basis for diagnosis | 80 | 52.7% (41.1, 65.3) | 63.2% (49.8, 76.6) |
| cM1 inferred from extent of disease variable | 1291 | 51.2% (48.3, 54.1) | 67.1% (64.1, 70.1) |
| cM1 inferred from PSA > 100 ng/ml | 655 | 47.8% (43.9, 51.8) | 65.3% (61.0, 69.5) |
| **Known cM0** | **7576** | **3.7% (3.3, 4.1)** | **13.8% (12.9, 14.9)** |
| cM0 inferred from extent of disease variable | 8544 | 2.1% (1.8, 2.5) | 10.1% (9.2, 10.9) |
| cM0 inferred from history of radical prostatectomy | 5208 | 0.8% (0.6, 1.1) | 3.9% (3.3, 4.7) |
| cM0 inferred based on low-risk disease at diagnosis | 2061 | 0.5% (0.3, 1.0) | 2.2% (1.5, 3.1) |
| **Known Gleason score ≥ 8** | **3588** | **19.1% (17.7, 20.5)** | **38.3% (36.0, 40.7)** |
| Gleason score ≥ 8 inferred from histologic grade 3 | 1946 | 23.9% (21.8, 26.1) | 48.2% (45.0, 51.5) |
| **Known Gleason score < 8** | **10,489** | **2.0% (1.7, 2.3)** | **7.8% (7.2, 8.6)** |
| Gleason score < 8 inferred from histologic grade 1 | 1634 | 1.9% (1.3, 2.7) | 12.2% (10.3, 14.5) |
| Gleason score < 8 inferred from histologic grade 2 | 3211 | 5.6% (4.8, 6.6) | 21.6% (19.6, 23.7) |

*PSA: Prostate-specific antigen*

## Figures

### Figure S1

Annual proportion of missing data for key variables used to **(A)** classify aggressive prostate cancer using the PC3 definition or **(B)** apply clinical assumptions to infer values for features used in aggressivity classification. Panel **(C)** illustrates the proportion of missing data after data consolidation using clinical assumptions with PPV_inferred_ > 95%.

### Figure S2

Cumulative prostate cancer-specific mortality among cases diagnosed during 2013–2022, stratified by aggressivity **(A)** classified using the Prostate Cancer Cohort Consortium (PC3) definition, and **(B)** among newly classified prostate cancer cases after applying clinical assumptions with PPV_inferred_ > 95%. Only 15 cases were newly classified as aggressive, and these were excluded from panel B.

### Figure S3

Cumulative prostate cancer-specific mortality among patients with or assumed to have/not have regional lymph node metastasis at diagnosis based on **(A)** known cN1 status, **(B)** inferred cN1 status from the extent of disease variable, **(C)** known cN0 status, **(D)** inferred to have cN0 status based on having low-risk disease, **(E)** inferred cN0 status from the extent of disease variable.

### Figure S4

Cumulative prostate cancer-specific mortality among patients with or without distant metastasis at diagnosis based on **(A)** known cM1 status, or **(B)** inferred cM1 status based on the basis for diagnosis variable, **(C)** the extent of disease variable, or **(D)** a prostate-specific antigen (PSA) > 100 ng/ml; **(E)** known cM0 status, or **(F)** inferred cM0 status based on the extent of disease variable, **(G)** a history of radical prostatectomy (RP), or **(H)** being diagnosed with low-risk disease.

### Figure S5

Cumulative prostate cancer-specific mortality among patients known or assumed to have/not have a fixed tumour or tumour invading adjacent structures at diagnosis based on **(A)** known cT4 status, **(B)** inferred cT4 status from the extent of disease variable, **(C)** known < cT4 status, **(D)** inferred < cT4 status from the extent of disease variable.

### Figure S6

Cumulative prostate cancer-specific mortality among patients with a known or inferred Gleason score: **(A)** known Gleason score ≥ 8, **(B)** inferred Gleason score ≥ 8 based on histologic grade 3 disease, **(C)** known Gleason score < 8, or **(D)** inferred Gleason score < 8 based on histologic grade 1 disease, **(E)** histologic grade 2 disease.

### Figure S7

Upset plot showing the frequency of cases inferred to have cN0 or cN1 status based on the clinical assumptions validated in this study (n = 9979). Labels on the bottom left of the plot describe the clinical assumptions validated. The bar plot shows the frequency of observed cases classified by various sets of clinical assumptions. These sets are indicated under the bar plot using various shapes, sometimes connected by a vertical line. Cases inferred as cN0 are marked in open shapes and cN1 in closed shapes.

### Figure S8

Upset plot showing the frequency of cases inferred to have cM0 or cM1 status based on the clinical assumptions tested in this study (n = 12,708). Labels on the bottom left of the plot describe the clinical assumptions included in the plot. The bar plot shows the frequency of observed cases classified by various sets of clinical assumptions. These sets are indicated under the bar plot using various shapes, sometimes connected by a vertical line. Cases classified as cM0 are marked in open shapes, and cM1 in closed shapes.

### Figure S9

Cumulative prostate cancer-specific mortality among patients with <cT3, Gleason score <8, and cM0 disease at diagnosis, stratified by whether cN information was missing (cNX or N-missing) or available (cN0/N1) at the Cancer Registry of Norway.
